# Magnesium isoglycyrrhizinate prevents the nonalcoholic hepatic steatosis via regulating energy homeostasis

**DOI:** 10.1111/jcmm.15230

**Published:** 2020-05-15

**Authors:** Wenjiao Jiang, Shiyu Xu, Huijie Guo, Li Lu, Jie Liu, Guangji Wang, Kun Hao

**Affiliations:** ^1^ Key Laboratory of Drug Metabolism and Pharmacokinetics China Pharmaceutical University Nanjing China

**Keywords:** glutamate, lipid metabolism, non‐alcoholic fatty liver disease, TCA cycle, TLR4

## Abstract

Non‐alcoholic fatty liver disease is a public health problem worldwide associated with high morbidity and hepatic steatosis, but no effective therapeutic interventions. Magnesium isoglycyrrhizinate (MGIG), a derivative of an active component of *Glycyrrhiza glabra*, is widely used for the treatment of inflammatory liver diseases due to its potent anti‐inflammatory and hepatoprotective activities. Hence, this study aimed to study the effects of MGIG on hepatic steatosis in mice fed a high‐fat diet (HFD). Oil Red O staining and transmission electron microscopy revealed a decrease in lipid accumulation in the liver after MGIG treatment along with improved mitochondrial ultramicrostructures. Metabonomic analysis demonstrated that MGIG intervention increased glutamate utilization in mitochondria by promoting the uptake of glutamate into the tricarboxylic acid (TCA) cycle. The NAD^+^/NADH ratio and the expression of other lipid‐metabolism‐related genes were increased in MGIG‐treated livers. Transcriptome sequencing showed that the expression of TLR4, an isoform of the innate immunity Toll‐like receptors (TLRs), was significantly decreased after MGIG treatment, suggesting a link between the anti‐inflammatory effects of MGIG and its suppression of lipidation. Our results reveal the potent effects of MGIG on lipid metabolism and suggest that hepatic TLR4 might be a crucial therapeutic target to regulate energy homeostasis in hepatic steatosis.

## 1 INTRODUCTION

Non‐alcoholic fatty liver disease (NAFLD) is a growing public health problem worldwide that is linked to high morbidity.^1^ Occurring in the absence of alcohol consumption, it is a common metabolic disease characterized by fat accumulation, non‐alcoholic steatohepatitis, fibrosis and irreversible cirrhosis.^2^ NAFLD is also treated as a metabolic syndrome that is strongly associated with metabolic diseases such as insulin resistance, hypertension, dyslipidaemia and obesity.^3^ A high‐fat diet (HFD) and inflammation serve as inducers to contribute to the progression of NAFLD.^4^ However, the underlying mechanism of NAFLD remains unclear.

Liver steatosis is attributed to an imbalance between lipid input (lipid uptake and lipogenesis) and output (lipid export and oxidation).^5^ The increased levels of free fatty acids (FFAs) in hepatocytes lead to blocked β‐oxidation, further aggravating lipid accumulation. Therefore, the HFD method is used to induce liver steatosis. Studies have demonstrated that inflammatory cytokines regulated by nuclear factor‐kappa B (NF‐κB) are involved in the development of NAFLD.^6^, ^7^ Overexpression of NF‐κB has been observed in NAFLD patients and mice that were fed HFD.^8^ 5′‐adenosine monophosphate (AMP)‐activated protein kinase (AMPK) is a serine/threonine kinase that maintains energy homeostasis and drives the downstream activation of peroxisome proliferator‐activated receptor alpha (PPARα) and PPAR‐gamma co‐activator‐1 alpha (PGC‐1α).^9^ AMPK‐mediated energy metabolism promotes fatty acid oxidation in HFD‐fed mice.^10^ Fatty liver disease can be attributed to a variety of major lipogenesis‐controlling factors, such as sterol regulatory element‐binding protein‐1c (SREBP‐1c). SREBP‐1c is induced by insulin and is responsible for the regulation of critical enzymes in fatty acid synthesis, including fatty acid synthase (FASN) and acetyl‐CoA carboxylase 1 (ACC1).^11^

Magnesium isoglycyrrhizinate (MGIG), a magnesium salt of 18‐α glycyrrhizic acid, has been designed based on the active component of the medicinal herb *Glycyrrhiza glabra*, also known as liquorice. At present, MGIG has been widely used for the treatment of inflammatory liver diseases due to its potent anti‐inflammatory and hepatoprotective activities.^12^, ^13^, ^14^ However, there are no reports on the in vivo effects of MGIG on non‐alcoholic hepatic steatosis. Hence, the present study aimed to investigate the pharmacological effects of MGIG on HFD‐induced hepatic steatosis in mice and explore the underlying mechanism.

## 2 MATERIALS AND METHODS

### 2.1 Reagents

Magnesium isoglycyrrhizinate injection was purchased from Chia Tai Tianqing Pharmaceutical Group Co., Ltd. Atorvastatin was purchased from Sigma‐Aldrich. Alanine aminotransferase (ALT), aspartate aminotransferase (AST), total cholesterol (TC), triglyceride (TG), low‐density lipoprotein cholesterol (LDL‐C) and high‐density lipoprotein cholesterol (HDL‐C) detection kits were supplied by the Nanjing Jiancheng Bioengineering Institute of Nanjing. Interleukin‐1 beta (IL‐1β), IL‐6 and tumour necrosis factor‐alpha (TNF‐α) enzyme‐linked immunosorbent assay (ELISA) kits were purchased from Shanghai Excell Biological Technology Co., Ltd. The colorimetric nicotinamide adenine dinucleotide (oxidized form/reduced form, NAD^+^/NADH) assay kit was obtained from BioVision Inc. All the antibodies were obtained from Cell Signaling Technology.

### 2.2 Animals

Male C57BL/6 mice (6 weeks old) were purchased from Shanghai SIPPR‐BK Lab Animal Co. Ltd. During the study, the animals were kept in an air‐conditioned room at 23 ± 2°C with a 12‐hour light/dark cycle. Additionally, animals were provided with water and food ad libitum and allowed to acclimate to the conditions of the animal centre for a week prior to the start of experiments. All animal studies were approved by the Animal Ethics Committee of China Pharmaceutical University and conducted in accordance with the National Institutes of Health Guidelines for the Care and Use of Laboratory Animals.

### 2.3 Experimental design

Animals used in the study were randomly allocated to five groups: control group, model group treated with HFD, atorvastatin group treated with HFD + atorvastatin (20 mg/kg, intragastric [i.g.]), MGIG 10 mg/kg group treated with HFD + MGIG (10 mg/kg, intraperitoneal [i.p.]), and MGIG 30 mg/kg group treated with HFD + MGIG (30 mg/kg, i.p.). The animals were fed a HFD prepared with the classic recipe (Hayek diet, Harlan [Teklad], TD88137) to mimic the human Western diet model for 12 weeks. The control animals consumed a standard diet during this period. From the 7th week onwards, the MGIG mice were intraperitoneally treated with MGIG (10 or 30 mg/kg) once a day for 6 weeks, and atorvastatin group (positive group) was intragastrically treated once a day with atorvastatin (20 mg/kg). During this period, the mice in the control and HFD groups received normal saline. At the end of the experiment, the animals were sacrificed and blood was immediately collected from the abdominal aorta. Sera were collected after centrifugation at 7000 *g* for 10 minutes at 4°C and stored at −80°C until analysis. Liver tissue was removed, washed with ice‐cold saline, and weighed. Parts of the livers were fixed in 10% neutral formalin for staining, while the remaining parts were harvested and stored at −80°C for the further analysis.

### 2.4 Biochemical assays

Serum levels of ALT and AST were examined to evaluate the severity of hepatic injury. The activities of ALT and AST in serum and levels of total cholesterol (TC), triglycerides (TG), low‐density lipoprotein cholesterol (LDL‐C), high‐density (HDL‐C) in serum and liver were detected with commercially available kits (Jiancheng Bioengineering Inc) according to the manufacturer's protocol. Glucose in serum was measured using the Glucocard^®^ and glucometer (Johnson & Johnson), according to the manufacturer's instructions.

### 2.5 Inflammatory cytokines

The levels of IL‐1β, IL‐6 and TNF‐α in serum and liver tissues were determined using an enzyme‐linked immunosorbent assay (ELISA) kit (Shanghai Excell Biological Technology Co., Ltd.), according to the manufacturer's instructions.

### 2.6 Nicotinamide adenine dinucleotide

Liver NAD evaluation was performed using a colorimetric NAD^+^/NADH assay kit (BioVision), according to the manufacturer's instructions.

### 2.7 Histopathological examination and Oil Red O staining

Hepatic tissues were removed for histological evaluation after the sacrifice, fixed in 10% (v/v) neutral‐buffered formalin for 48 hours. Briefly, the samples were dehydrated in graded alcohol and deparaffinized with xylene. After embedding in paraffin wax, the sections were sliced and stained with haematoxylin and eosin (H&E). Histopathological examination was performed under a light microscope in a blinded manner. For Oil Red O staining, livers embedded in freezing medium were cut into 5 μm sections with a freezing microtome (Leica). The frozen sections were then stained with Oil Red O, followed by counter‐staining with haematoxylin. The lipid droplets stained in red were visualized under a light microscope and recorded.

### 2.8 Transmission electron microscopy

For transmission electron microscopy (TEM), liver tissues were fixed in 2.5% (w/v) glutaraldehyde overnight, followed by post‐fixation with 1% (w/v) osmium tetroxide and embedment using epoxy resin. The tissue samples were sliced at a thickness of 50‐80 nm with an LKB Ultramicrotome, followed by incubation with uranyl acetate‐lead citrate. Ultramicrostructure associated with lipid droplets and mitochondrial morphology was observed with an electron microscope (Hitachi; H‐7650).

### 2.9 Quantitative reverse‐transcription polymerase chain reaction (qRT‐PCR)

Total RNA was extracted from liver tissues using TRIzol^™^ reagent (Invitrogen) and reverse transcribed to cDNA using the Primescript^™^ RT Master Mix Kit (Takara; RR036A) in accordance with the protocol. Real‐time polymerase chain reaction was performed using SYBR Green Supermix (Bio‐Rad); ^ΔΔ^Ct method was used for relative quantification. Relative gene expression was determined by normalizing expression of the different genes to that of glyceraldehyde 3‐phosphate dehydrogenase (GAPDH). The primer sequences used in our study are listed as follows:

*SREBP‐1c*: ATCGGCGCGGAAGCTGTCGGGGTAGCGTC (F), ACTGTCTTGGTTGTTGATGAGCTGGAGCAT (R);
*FASN*: GGAGGTGGTGATAGCCGGTAT (F), TGGGTAATCCATAGAGCCCAG (R);
*ACC1*: ATGGGCGGAATGGTCTCTTTC (F), TGGGGACCTTGTCTTCATCAT (R);
*SCD1*: TTCTTGCGATACACTCTGGTGC (F), CGGGATTGAATGTTCTTGTCGT (R);
*CD36*: ATGGGCTGTGATCGGAACTG (F), TTTGCCACGTCATCTGGGTTT (R);
*LXRα*: CAAGGGAGCACGCTATGTCTG (F), GGACACCGAAGTGGCTTGAG (R);
*PPARα*: AGAGCCCCATCTGTCCTCTC (F), ACTGGTAGTCTGCAAAACCAAA (R);
*PGC‐1α*:TATGGAGTGACATAGAGTGTGCT (F), CCACTTCAATCCACCCAGAAAG (R);
*AMPK*: TCTGAGGGGCACCAAGAAAC (F), GTGGGTGTTGACGGAGAAGAG (R);
*TLR2*: TCTAAAGTCGATCCGCGACAT (F), CTACGGGCAGTGGTGAAAACT (R);
*TLR4*: ATGGCATGGCTTACACCACC (F), GAGGCCAATTTTGTCTCCACA (R);
*TLR9*: ACAACTCTGACTTCGTCCACC (F), TCTGGGCTCAATGGTCATGTG (R);
*Myd88*: AGGACAAACGCCGGAACTTTT (F), GCCGATAGTCTGTCTGTTCTAGT (R);
*NLRP3*: ATTACCCGCCCGAGAAAGG (F), TCGCAGCAAAGATCCACACAG (R);
*ASC*: CTTGTCAGGGGATGAACTCAAAA (F), GCCATACGACTCCAGATAGTAGC (R);
*Caspase‐1*: AATACAACCACTCGTACACGTC (F), AGCTCCAACCCTCGGAGAAA (R);
*HMGB1*: GCTGACAAGGCTCGTTATGAA (F), CCTTTGATTTTGGGGCGGTA (R);
*GAPDH*: AGGTCGGTGTGAACGGATTTG (F), TGTAGACCATGTAGTTGAGGTCA (R).

### 2.10 Western blotting

The liver tissues were homogenized, washed with phosphate‐buffered saline (PBS), and incubated in lysis buffer (Nanjing KeyGEN Biotech Co., Ltd.)．The hepatic protein concentration was quantitated with a bicinchoninic acid protein assay kit (Beyotime Institute of Biotechnology) prior to incubation with loading buffer and 2‐mercaptoethanol at 100°C for 5 minutes. The extracted protein was separated by sodium dodecyl sulphate‐polyacrylamide gel electrophoresis and transferred onto polyvinylidene difluoride membranes (Millipore). Non‐specific binding sites were blocked with 5% (w/v) non‐fat dry powdered milk in Tris‐buffered saline containing Tween 20 (TBST) and then incubated overnight with appropriate antibodies at 4°C. The blots were washed and incubated with the appropriate horseradish peroxidase‐conjugated secondary antibodies for 2 hours at room temperature. Specific bands were visualized by enhanced chemiluminescence (Bio‐Rad) using Image Lab Software (Bio‐Rad; version 6.0).

### 2.11 Metabonomic analysis

Livers isolated from each group were homogenized and mixed with 80% (v/v) methanol solution containing 15 μg/mL of 5‐^13^C‐glutamine (Cambridge Isotope Laboratories) as the internal standard. After centrifugation at 16 000 *g* for 10 minutes, the supernatant was collected and evaporated to dryness. The residue was dissolved, centrifuged and prepared for liquid chromatography‐quadrupole/time‐of‐flight mass spectrometry (LC‐Q/TOF‐MS)‐based metabolomics. Subsequent metabonomic analysis was performed as previously described.^15^ MultiQuant 2.0 analytical software (AB SCIEX, USA) was used for data analysis, and the quantification of samples was carried out with the peak area under protein concentration correction. The overall difference between groups was investigated with partial least squares discriminant analysis using SIMCA‐P software (Sweden). Furthermore, 3D sparse partial least squares discriminant analysis and heatmaps were generated at MetaboAnalyst (http://www.metaboanalyst.ca).

### 2.12 Transcriptome sequencing

Total RNA was extracted from liver samples with TRIzol^™^ reagent (Invitrogen) and quantified with Qubit (Life; Q32855). After obtaining mRNA by polyA RNA selection, a transcriptome sequencing (RNA‐Seq) library was constructed and sequenced on the Illumina HiSeq platform using VAHTS^™^ mRNA‐seq V2 Library Prep Kit for Illumina according to the manufacturer's protocol. After FAST‐QC evaluation, heatmaps were generated using the HISAT2 software program. Gene ontology (Go) term enrichment analysis was conducted for differential genes using the clusterProfiler software. Only *P* values <.05 were considered to be significantly enriched in the GO enrichment analysis.

### 2.13 Statistical analysis

Data have been expressed as mean values ± standard deviation (SD). Differences between groups were analysed by one‐way analysis of variance (ANOVA) with Tukey's multiple comparison test using GraphPad Prism 7.0a software. A *P* value <.05 was considered to be significant.

## 3 RESULTS

### 3.1 Effects of MGIG on liver steatosis

**FIGURE 1:**
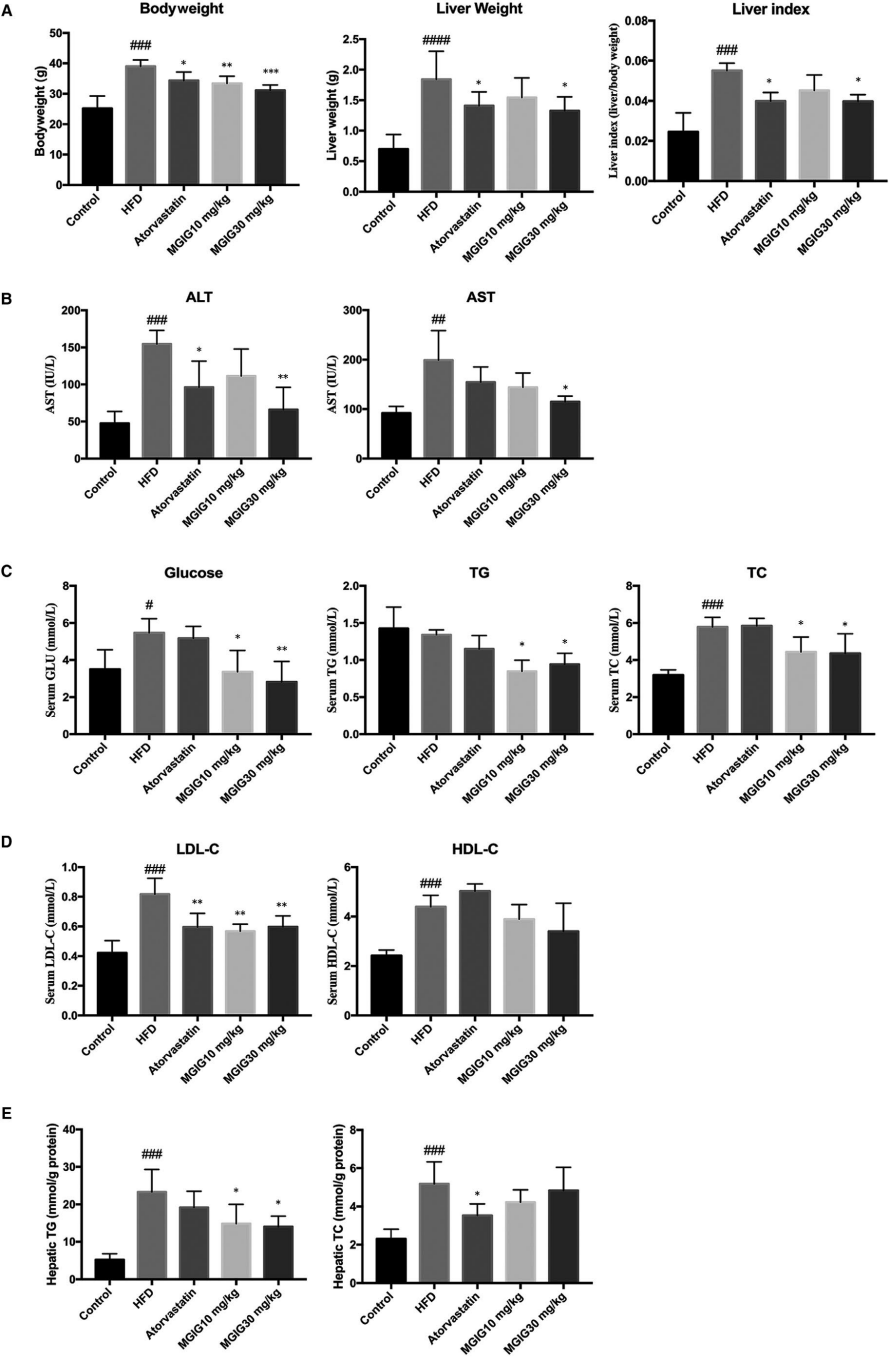
The protective effect of magnesium isoglycyrrhizinate (MGIG) treatment on hepatic injury caused by high‐fat diet (HFD). The liver steatosis was induced by a HFD for 12 wk. The MGIG groups were intraperitoneally treated with MGIG (10 or 30 mg/kg) once a day for 6 wk since 7th week. The bodyweight, liver weight and the ratio of liver weight/bodyweight (A). The activities of alanine aminotransferase (ALT) and aspartate aminotransferase in serum (AST; B). The serum levels of glucose, triglyceride (TG) and total cholesterol (TC; C). The serum levels of low‐density lipoprotein cholesterol (LDL‐C) and high‐density lipoprotein cholesterol (HDL‐C; D). The hepatic levels of triglyceride (TG) and total cholesterol (TC; E). The data were presented as means ± SDs. Compared with Control group: ^#^ *P* < .05, ^##^ *P* < .01, ^###^ *P* < .001. Compared with Model group: *P < .05, **P < .01, ***P < .001 (n = 6)

**FIGURE 2:**
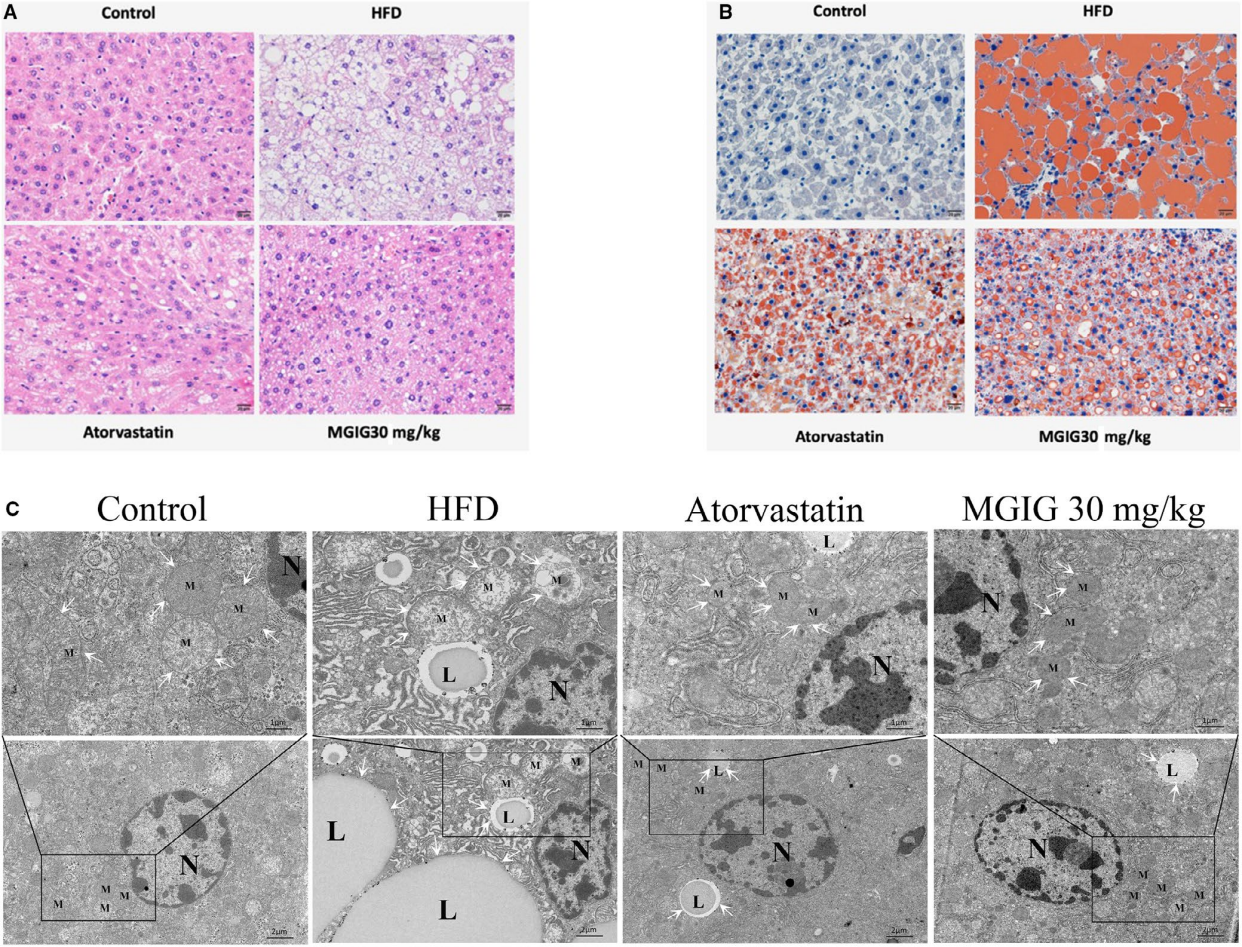
Effects of magnesium isoglycyrrhizinate (MGIG) treatment on high‐fat diet (HFD)‐induced lipid accumulation in livers. Representative photographs of histopathologic changes are presented (A). Representative photographs of Oil Red O staining are presented (B). Ultramicrostuctures of mitochondria and lipid droplets in livers are presented by transmission electron microscopy (C). The data were presented as means ± SDs. Compared with Control group: ^#^ *P* < .05, ^##^ *P* < .01, ^###^ *P* < .001. Compared with Model group: *P < .05, **P < .01, ***P < .001 (n = 3)

First, we investigated the protective effect of MGIG on HFD‐induced liver injury in mice. These data are presented in Figure 1 and show that the HFD contributed to a considerable increase in bodyweights, transferase activities and dyslipidaemia. In contrast, the treatment with atorvastatin and MGIG (30 mg/kg) effectively suppressed obesity and hepatic oedema caused by HFD, as evident by the decrease in bodyweight and improvement in the liver index, a ratio of liver weight to bodyweight. Atorvastatin and MGIG treatment were accompanied by the reversal of the increase in transferases (ALT, AST) activity. MGIG intervention significantly improved dyslipidaemia, showing marked decrease in levels of TG, TC and LDL‐C, but not of HDL‐C. Histopathological analysis confirmed the hepatoprotective effect of MGIG, with fewer hepatic vacuoles and less inflammatory infiltration being observed in the MGIG‐treated mice than in HFD model mice (Figure 2A).

The severity of lipidation in the liver was also assessed. As shown in Figure 1E, MGIG treatment markedly attenuated the HFD‐stimulated rise in hepatic TG levels, but not in hepatic TC levels, suggesting that MGIG could ameliorate HFD challenge‐induced triglyceride deposition, but not cholesterol stress. Consistent with the trend observed for the hepatic TG content, an apparent suppression of lipid accumulation by MGIG was observed with Oil Red O staining (Figure 2B). TEM performed in liver samples to observe ultramicrostructures revealed that the number of intracytoplasmic lipid droplets in MGIG samples was closer to that seen in control samples and was a sharp contrast to the high number of lipid droplets in HFD samples (Figure 2C). Thus, MGIG showed an ameliorative effect of MGIG on HFD‐induced lipidation in the liver.

### 3.2 MGIG improved energy metabolism by regulating the uptake of glutamate into the tricarboxylic acid cycle

Intriguingly, the effect of MGIG on mitochondrial morphology observed during the examination of lipid droplets drew our attention to energy metabolism in MGIG‐treated mice. As shown in Figure 2C, in the control group, the ultramicrostructure of endoplasmic reticulum was clear and recognizable, and the abundant cytochondriome appeared with a complete crista structure. However, the HFD group showed the presence of abnormal ultramicrostructures to some extent, as evidenced by a high number of fat vacuoles and the mitochondria in the cytoplasm showing an obvious distension, with disrupted crista structure. MGIG treatment attenuated the aforementioned HFD‐induced changes as compared to the model group, especially with respect to the integrity of mitochondria. These data demonstrate that MGIG attenuated HFD‐induced lipotoxicity by reducing mitochondrial damage, which was consistent with the findings of previous study.^16^

**FIGURE 3:**
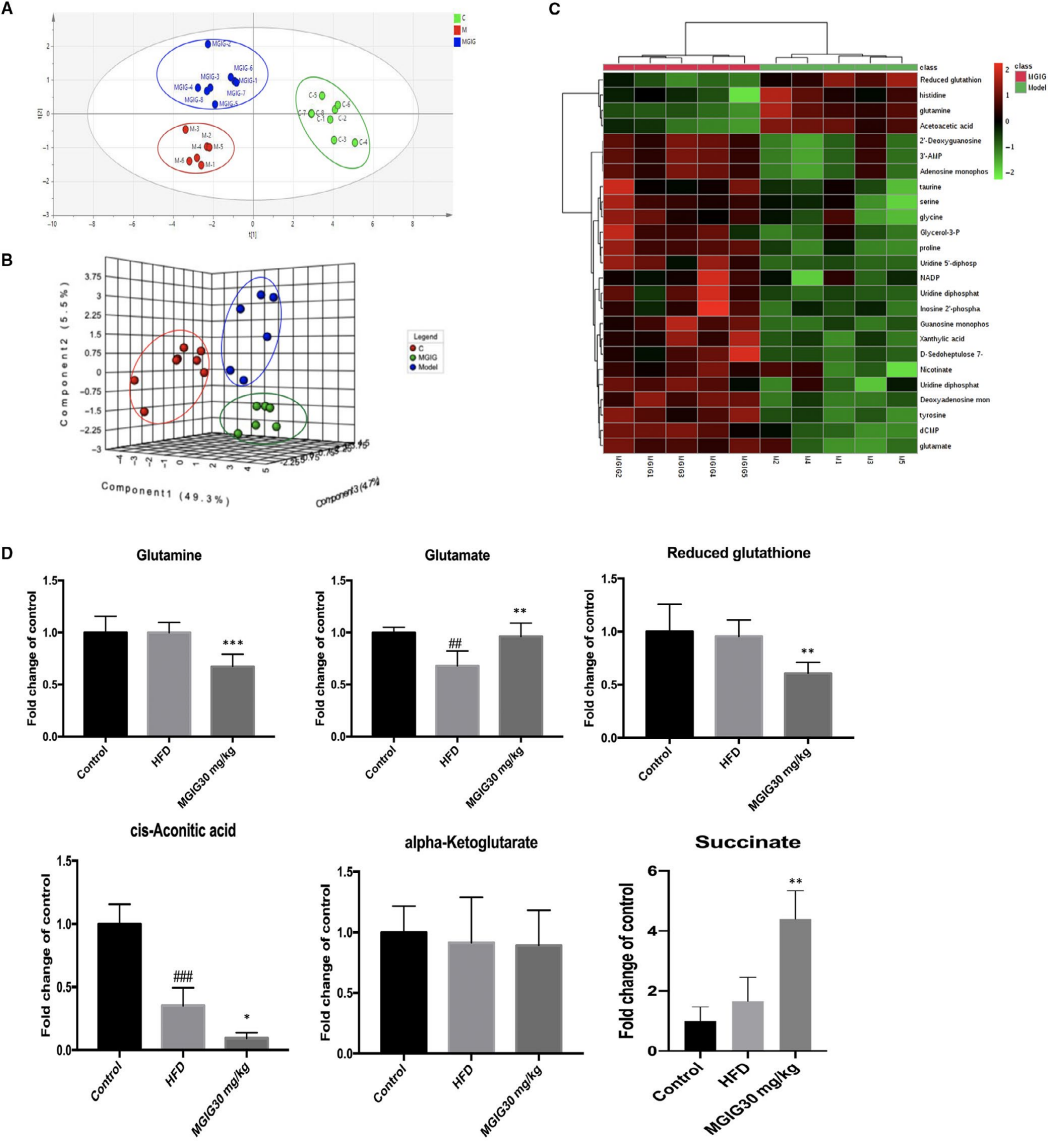
Magnesium isoglycyrrhizinate (MGIG) improves energy metabolism via regulating the uptake of glutamate into tricarboxylic acid (TCA) cycle. Livers of each group were homogenized and mixed with 80% methanol solution containing 5‐^13^C‐glutamine for the following metabonomic analysis. The PLS‐DA plot of control, HFD model and MGIG groups for liver data (A). The sPLS‐DA 3D plot of control, HFD model and MGIG groups for liver data (B). Heatmaps exhibiting metabolites in livers of MGIG mice and HFD mice (C). The metabolites associated with glutamate and TCA cycle metabolic pathways in liver tissues after MGIG treatment (D). The data were presented as means ± SDs. Compared with Control group: ^#^ *P* < .05, ^##^ *P* < .01, ^###^ *P* < .001. Compared with Model group: *P < .05, **P < .01, ***P < .001 (Control: n = 8, HFD: n = 6; MGIG: n = 8)

Given the improvement in mitochondrial structure in MGIG samples, metabonomic analysis was performed to evaluate whether MGIG contributed to the change in mitochondrial morphology by affecting the metabolic pattern. A metabolic abnormality was observed under HFD conditions, and MGIG therapy led to a significant reversion in the HFD‐induced global metabolomics of liver tissues. Principal component analysis and heatmaps revealed distinguishing features among the different groups, reflecting significant metabolic shifts in the HFD model and with MGIG intervention (Figure 3A‐C). Among the altered metabolites (Figure 3D), there were significant changes in metabolites associated with glutamate metabolism and the tricarboxylic acid (TCA) cycle in the livers of mice treated with MGIG (30 mg/kg). Levels of glutamate were higher, whereas those of glutamate precursor, glutamine, were lower in MGIG‐treated livers in comparison with HFD livers, suggesting that MGIG promoted the conversion of glutamine into glutamate. The MGIG‐induced increased levels of glutamate could lead to increased formation of alpha‐ketoglutarate, as evidenced by the increased level of succinate, a subsequent metabolite of the TCA cycle. Furthermore, the decrease in glutathione levels indicates that MGIG might divert glutamate to the TCA cycle at the expense of glutathione synthesis, which could explain the enhanced energy metabolism in MGIG‐treated livers. Hence, these data suggest that the diversion of the increased glutamate levels away from glutathione synthesis towards the TCA cycle results in enhanced energy metabolism and lower glutathione levels in MGIG‐treated livers.

### 3.3 Effects of MGIG on nicotinamide adenine dinucleotide

**FIGURE 4:**
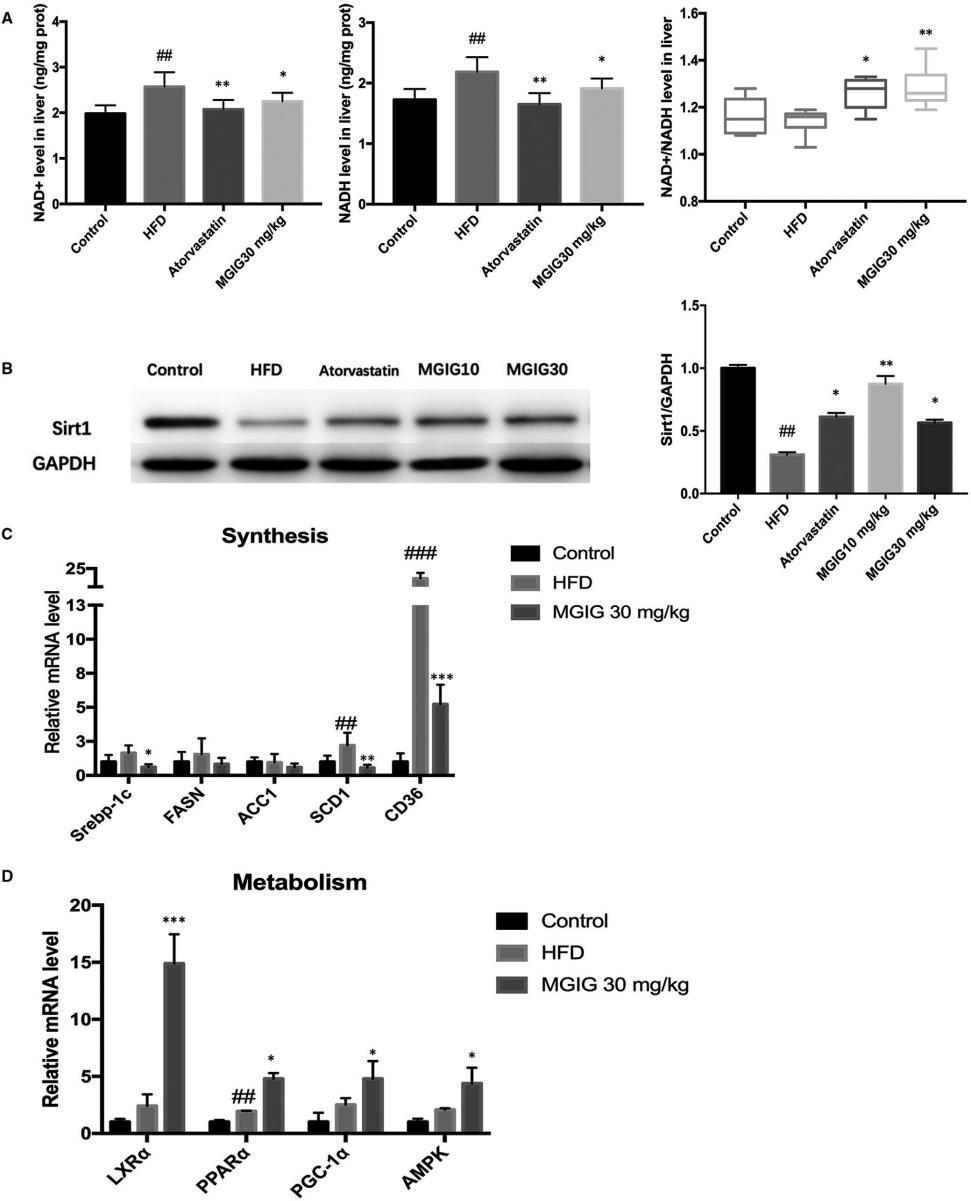
Magnesium isoglycyrrhizinate (MGIG) treatment improved hepatic steatosis by promoting lipid metabolism. The hepatic levels of NAD+, NADH and NAD+/NADH ratio (A). The protein expression of deacetylase sirtuin 1 (Sirt1) and its quantification data in liver samples (B). The lipid synthesis‐related genes expressions (C). The lipid metabolism‐related genes expressions (D). The data were presented as means ± SDs. Compared with Control group: ^#^ *P* < .05, ^##^ *P* < .01, ^###^ *P* < .001. Compared with Model group: *P < .05, **P < .01, ***P < .001 (n = 6)

To further explore the influence of MGIG in mitochondrial redox reactions, we assessed the mitochondrial marker, nicotinamide adenine dinucleotide (NAD^+^), an essential metabolite and a common hydrogen carrier in the mitochondrial respiratory chain. As the conversion of NAD^+^ into NADH plays a critical role in the oxidation of fatty acids and amino acids in the mitochondria, the ratio of NAD^+^ to NADH is universally applied for the diagnosis of mitochondrial activity.^17^ In our study, the levels of NAD^+^ and NADH in the livers of MGIG‐treated mice were notably lower when compared to those of the HFD model mice. Despite this difference, the NAD^+^/NADH ratio was clearly elevated after MGIG administration, a change that was also observed in the atorvastatin group (Figure 4A). These data indicate that MGIG and atorvastatin both have beneficial effects on lipid metabolism, namely through NAD^+^ metabolism and the adjustment of the overall redox balance under HFD conditions. An altered NAD^+^/NADH ratio affects directly the deacetylase, sirtuin 1 (Sirt1), which coordinates a metabolic switch from glucose to fatty acid metabolism.^18^, ^19^ As shown in Figure 4B, MGIG and atorvastatin groups showed a reversal of the HFD‐induced decrease in Sirt1 levels, which is consistent with the altered NAD^+^/NADH ratio.

### 3.4 Effects of MGIG on lipid metabolism‐related gene expression in liver

To explore the mechanism underlying the effects of MGIG on liver steatosis, lipid metabolism‐related gene expression was analysed by qPCR. As depicted in Figure 4C, HFD stimulation markedly up‐regulated the expression of stearoyl‐CoA desaturase‐1 (SCD1) and cluster of differentiation (CD36 or fatty acid translocase (FAT)), the genes involved in lipidation. MGIG treatment significantly suppressed lipid synthesis gene transcription, decreasing mRNA levels of SREBP‐1c, SCD1 and CD36, but increasing mRNA levels of liver X receptor alpha (LXRα), AMPK, PPARα and PGC‐1α as compared to those of the model group, which was consistent with the previous data on the effects of MGIG on lipid metabolism (Figure 4D).

### 3.5 Anti‐inflammatory effects of MGIG

**FIGURE 5:**
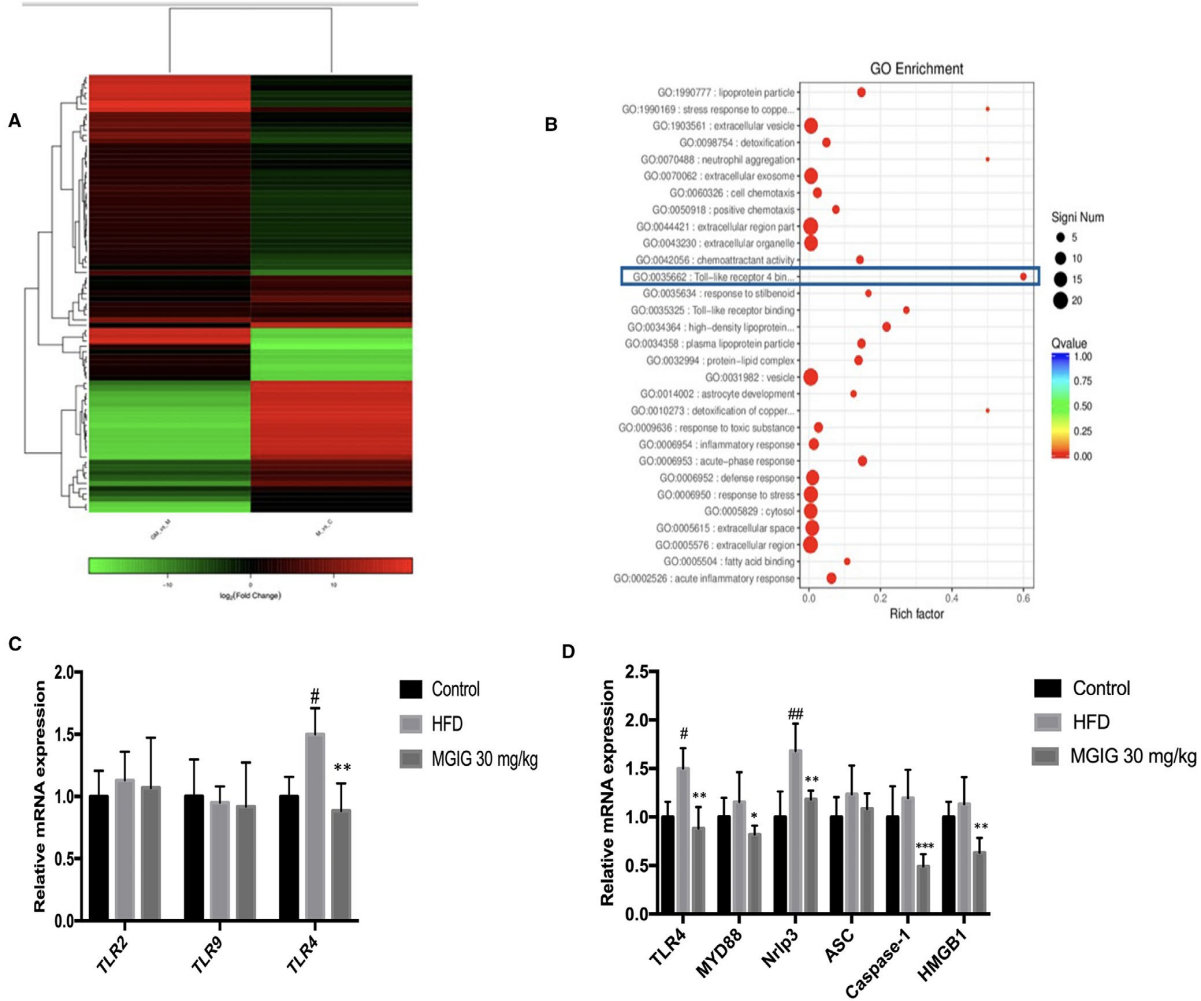
Inhibition in Toll‐like receptor 4 binding pathway was associated with the protective effect of magnesium isoglycyrrhizinate (MGIG) intervention. Transcriptome profiling was conducted to further find the differential gene between MGIG mice and high‐fat diet (HFD) mice. Heatmap exhibiting the reversion of MGIG treatment in gene expression (A). The left column represents the differential genes when MGIG vs HFD model group, and the right column represents the differential genes when HFD model vs control group. The clustering of significant differential genes between MGIG group and HFD model group to find the pathways these genes involved in (B). The gene expressions of Toll‐like receptors (TLRs) isoforms were evaluated (C). The expressions of TLR4‐related genes were assessed (D). The data were presented as means ± SDs. Compared with Control group: ^#^ *P* < .05, ^##^ *P* < .01, ^###^ *P* < .001. Compared with Model group: *P < .05, **P < .01, ***P < .001 (n = 6)

AMPK is a crucial regulator of energy metabolic homeostasis and inflammation.^20^ MGIG is a magnesium salt of 18‐α glycyrrhizic acid, an active component extracted from the root of Radix Glycyrrhizae, has been widely utilized in the clinic for its anti‐inflammatory activity.^21^, ^22^ Therefore, transcriptome profiling was conducted to further explore the potential mechanism responsible for the protective effects of MGIG on HFD‐induced liver steatosis. As shown in the Figure 5A, the samples from different groups were well separated and the treatment with MGIG reversed the HFD‐induced regulation changes in gene expression. An examination of the clustering of the differential genes of the MGIG and HFD model group revealed that the largest shift in these genes was in the Toll‐like receptor 4 binding pathway (Figure 5B). Thus, RNA sequencing uncovered the MGIG‐related differences in innate immunity Toll‐like receptor 4 activation under HFD conditions.

Next, qPCR was performed to study the hepatic gene expression of the innate immune system Toll‐like receptor (TLR) isoforms, including TLR2, TLR4 and TLR9. Only the TLR isoform, TLR4, responded to stimulation with HFD and MGIG. TLR4 gene expression was notably up‐regulated in response to HFD challenge, but was attenuated by the following MGIG intervention (Figure 5C). The same trend was observed in the expression of TLR4‐related genes, including high mobility group box protein 1 (HMGB1), myeloid differentiation primary response 88 (Myd88), Nacht domain leucine‐rich repeat family pyrin domain‐containing 3 (Nrlp3), apoptosis‐associated speck‐like protein containing a caspase‐recruitment domain (CARD) (ASC), and caspase‐1, demonstrating the involvement of Toll‐like receptor 4 (TLR4) signalling in the regulation of energy metabolic homeostasis under conditions of HFD stimulation (Figure 5D).

**FIGURE 6:**
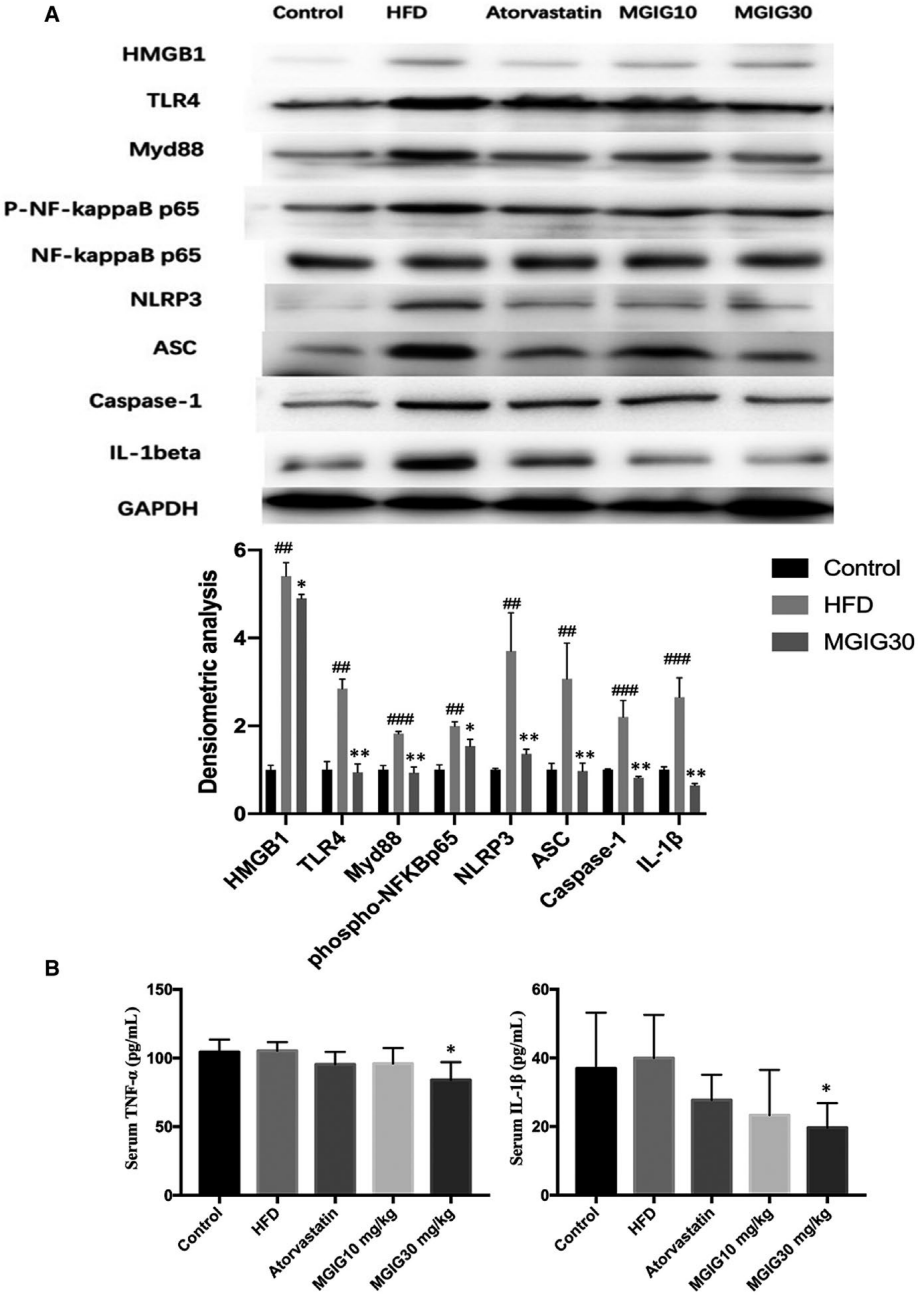
The anti‐inflammatory property of magnesium isoglycyrrhizinate (MGIG) accounts for its resistance to lipidation. The effects of MGIG on the protein expressions of HMGB1, TLR4, Myd88, the phosphorylation of NF‐κBp65 and activation of Nlrp3 inflammasome in liver tissues (A). The levels of TNF‐α, IL‐1β in serum (B). The data were presented as means ± SDs. Compared with Control group: ^#^ *P* < .05, ^##^ *P* < .01, ^###^ *P* < .001. Compared with Model group: *P < .05, **P < .01, ***P < .001 (n = 6)

Subsequent experiments focused on the effects of MGIG intervention on the levels of proteins of the TLR4/NF‐κB/Nlrp3 signalling pathway in HFD‐induced hepatic steatosis. As shown in Figure 6A, HFD contributed to the increase in protein levels of TLR4, the NF‐κBp65 phosphorylation, and Nlrp3 inflammasome activation in liver tissues. Administrations of atorvastatin and MGIG effectively prevented HFD‐triggered protein‐related changes described above. These findings suggest that MGIG exerted its protective effect on energy metabolic homeostasis by suppressing TLR4/NF‐κB/Nlrp3 pathway‐mediated inflammation. We then evaluated the anti‐inflammatory effects of MGIG by measuring inflammatory cytokine levels using ELISA kits. As illustrated in Figure 6B, MGIG (30 mg/kg) therapy significantly suppressed the secretions of TNF‐α and IL‐1β into the serum. These results indicate that treatment with MGIG could inhibit the inflammation by regulating TLR4 signalling in HFD‐stimulated mice with NAFLD.

## 4 DISCUSSION

Acetyl‐CoA carboxylase 1 and FASN play key roles in the synthesis of fatty acids to regulate hepatic lipid synthesis.^23^ However, the expression of ACC1 and FASN are influenced by SREBP‐1c, which is activated in response to insulin levels. Therefore, SREBP‐1c, which is synthesized as a precursor in the endoplasmic reticulum, is the key regulator of hepatic de novo lipid synthesis.^24^ Thus, mature SREBP‐1c functions as an activated transcription factor regulating the expression of enzymes involved in hepatic lipogenesis such as FASN and ACC1.^25^ A significant increase in hepatic levels of SREBP‐1c and its target lipid synthesis enzymes, FASN and ACC, is observed in NAFLD patients as well as in HFD‐fed rodents, which is followed by a large amount of TG deposition.^26^, ^27^ Our study suggests that the MGIG could be used to correct metabolic disorders that would benefit from the suppression of the levels of SREBP‐1c and its downstream targets, SCD1 and FAT (CD36).

In addition, significant changes occurred in the mediators of lipid metabolism after MGIG treatment. AMPK is a sensitive regulator of energy metabolism, stimulating energy production from glucose and lipids while simultaneously suppressing energy consumption. Recent studies reported a decrease in AMPK expression in the HFD‐induced hepatic steatosis mice, indicating a role for AMPK in the pathogenesis of NAFLD.^28^, ^29^ As a crucial downstream target of AMPK, PGC‐1α is directly activated by phosphorylated AMPK as a transcriptional regulator of mitochondrial and oxidative metabolic programmes.^30^ The subsequent deacetylation of PGC‐1α causes it to co‐activate PPARα complexes, thus controlling the transcription of several metabolic genes.^31^ The PPAR family consists of members, α, β and γ, that are encoded by genes with distinct tissue distribution patterns.^32^ PPARα, a PPAR isoform predominantly expressed in the liver, is a ligand‐activated transcription factor that regulates the genes involved in FFA metabolism in the liver.^33^ Recent observations pointed out that a PPAR‐α knockdown mouse suffered from a severe disorder in hepatic FFA oxidation, affecting processing and causing hepatic lipid accumulation as well as dyslipidaemia.^34^ In our study, we found that NAD^+^‐dependent Sirt1 might be responsible for up‐regulation of AMPK/PGC‐1α/ PPARα genes to promote lipid oxidation.

Nuclear factor‐kappa B, a mediator associated with inflammation, functions as a primary mediator in steatohepatitis.^35^ Previous studies have reported HFD‐induced activation of NF‐κB in hepatocytes as well as hepatic insulin resistance in fatty liver.^36^ Studies have also shown that the activation of Toll‐like receptor 4 (TLR4) signalling in liver parenchymal cells, accompanied with the translocation of NF‐κBp65 into the nucleus, is involved in the initiation of NAFLD.^37^ In particular, HMGB1 released from hepatocytes in response to FFA infusion serves as a positive mediator in TLR4/MyD88 activation and cytokine production. In addition, TNF receptor‐associated factor (TRAF) family member‐associated NF‐κB activator (TANK)‐binding kinase 1 (TBK1), a downstream effector of TLR4 signalling, plays a unique role in mediating bidirectional cross‐talk between energy sensing and inflammatory signalling pathways.^38^ Although many studies have proposed that there is a negative correlation between Sirt1 and TLR4, that is yet to be elucidated.^39^, ^40^

Consistent with previous studies, we found that MGIG might exert its ameliorative effect on energy metabolism by increasing the conversion of glutamate into α‐ketoglutarate for the TCA cycle influx, supporting the notion that higher glutamine uptake could contribute to increased TCA activity.^41^ However, there is little information about the effect of TLR4 on energy metabolism, warranting further researches. In conclusion, our study revealed that MGIG administration alleviated hepatic steatosis in mice fed with HFD, as evidenced by inhibition of lipidation, marked histopathological changes, and improved mitochondrial activity. Our data suggest that hepatic TLR4 could be a crucial target to modulate energy homeostasis in hepatic steatosis.

## CONFLICT OF INTEREST

The authors confirm that there are no conflicts of interest.

## AUTHOR CONTRIBUTIONS

Guangji Wang and Kun Hao contributed conception and design of the study; Wenjiao Jiang, Shiyu Xu and Huijie Guo performed the experiments; Wenjiao Jiang, Li Lu and Jie Liu performed the statistical analysis; Wenjiao Jiang and Kun Hao wrote the first draft of the manuscript. All authors read and approved the final manuscript.

## Data Availability

The data that support the findings of this study are available from the corresponding author upon reasonable request.
